# CD30 Is Dispensable for T-Cell Responses to Influenza Virus and Lymphocytic Choriomeningitis Virus Clone 13 but Contributes to Age-Associated T-Cell Expansion in Mice

**DOI:** 10.3389/fimmu.2017.01156

**Published:** 2017-09-25

**Authors:** Angela C. Zhou, Laura M. Snell, Michael E. Wortzman, Tania H. Watts

**Affiliations:** ^1^Faculty of Medicine, Department of Immunology, University of Toronto, Toronto, ON, Canada

**Keywords:** CD30, viral, influenza, lymphocytic choriomeningitis virus, T cells, age-dependent T-cell expansion

## Abstract

CD30 is a tumor necrosis factor receptor (TNFR) family member whose expression is associated with Hodgkin’s disease, anaplastic large cell lymphomas, and other T and B lymphoproliferative disorders in humans. A limited number of studies have assessed the physiological role of CD30/CD30 ligand interactions in control of infection in mice. Here, we assess the role of CD30 in T-cell immunity to acute influenza and chronic lymphocytic choriomeningitis virus (LCMV) clone 13 infection, two viral infections in which other members of the TNFR superfamily are important for T-cell responses. We show that CD30 is expressed on activated but not resting CD4 and CD8 T cells *in vitro*, as well as on regulatory T cells and marginally on T helper 1 cells *in vivo* during influenza infection. Despite this, CD4 and CD8 T-cell expansion in response to influenza virus was comparable in CD30^+/+^ and CD30^−/−^ littermates, with no discernable role for the pathway in the outcome of influenza infection. Similarly, during persistent infection with LCMV clone 13, CD30 plays no obvious role in CD4 or CD8 T-cell responses, the level of T-cell exhaustion or viral control. In contrast, in the steady state, we observed increased numbers of total CD4 and CD8 T cells as well as increased numbers of regulatory T cells in unimmunized older (~8 months) CD30^+/+^ but not in CD30^−/−^ age-matched littermates. Naive T-cell numbers were unchanged in the aged CD30^+/+^ mice compared to their CD30^−/−^ littermate controls, rather the T-cell expansions were explained by an increase in CD4^+^ and CD8^+^ CD44^mid-hi^CD62L^−^ effector memory cells, with a similar trend in the central memory T-cell compartment. In contrast, CD30 did not impact the numbers of T cells in young mice. These data suggest a role for CD30 in the homeostatic regulation of T cells during aging, contributing to memory T-cell expansions, which may have relevance for CD30 expression in human T-cell lymphoproliferative diseases.

## Introduction

CD30, a member of the tumor necrosis factor receptor (TNFR) superfamily that is expressed on B cells, NK cells, eosinophils, macrophages, and activated T cells, is perhaps best studied for its overexpression on Reed–Sternberg cells in lymphoma (1, 2). Its ligand, CD30L (CD153), can be detected on dendritic cells, macrophages, resting B cells, neutrophils, eosinophils, activated T cells, as well as a CD4^+^CD3^−^CD11c^−^ accessory cell implicated in the survival of CD4 memory Th2 cells (1–3). Stimulation of CD30 on T cells via agonistic anti-CD30 antibody or recombinant CD30L in the presence of anti-CD3 or antigen-primed dendritic cells can enhance T-cell activation, proliferation and cytokine production (4–7). CD30 expression is a hallmark of various pathological lymphoproliferations and has been associated with classical Hodgkin’s lymphoma, anaplastic large cell lymphomas, and primary cutaneous CD30^+^ T-cell proliferative disorders (8–10).

Much work has focused on the role of CD30 in CD4 T-cell responses *in vivo*. CD30, in synergy with another TNFR family member OX40, was reported to be crucial for the survival of Th2 CD4 memory cells necessary to provide help to B cells for memory antibody responses (3, 11, 12). Indeed, CD30^−/−^ mice were found to have defective memory antibody responses compared to wild-type C57BL/6 mice (12). Moreover, Th2 cells preferentially express CD30 and OX40 and interact with CD30L- and OX40L-expressing CD4^+^CD3^−^ accessory cells at the T–B cell border to help B-cell responses (3, 11). It is possible that these Th2 cells identified are in fact the more recently discovered T follicular helper cell subset that contributes to the formation and maintenance of germinal centers and B-cell responses (13). CD30 has also been implicated in the polarization of CD4 Th17 cells (14, 15) and to play a role in several CD4 T helper 1 (Th1) responses (16–18).

There is also evidence of a role for CD30 in CD8 T-cell activation and the maintenance of CD8 T-cell memory (19–21). CD30L^−/−^ mice have defective generation of long-term memory CD8 T cells following *Listeria* infection, particularly affecting central memory (20). In contrast, studies of VSV and murine CMV (MCMV) infection revealed no role for CD30 in either CD8 T-cell or antibody responses (21, 22). Pox viruses of murine and bovine origin are noted to encode a soluble CD30 homolog, which inhibits CD30L binding to its cellular receptor (23, 24). The finding that CD30 is a target for subversion by viruses (23, 24) suggests that CD30 signaling may be important in anti-viral immunity. In addition to viral immunity, the CD30–CD30L pathway is important for the clearance of mycobacterial infections by mediating IL-17A production by γδT cells, as shown through studies with CD30^−/−^ mice (25, 26).

Here, we address the role of CD30 in T-cell immunity to viral infection by assessing an acute localized infection with influenza A virus and a chronic systemic infection with lymphocytic choriomeningitis virus (LCMV) clone 13. Several TNFR family members have previously been shown to have non-redundant and significant impact on T-cell responses in these two infection models (27–34). Surprisingly, however, by comparing CD30-deficient mice to their littermate wild-type controls, we found that CD30 appears to be completely dispensable for CD4 and CD8 T-cell responses to these two viruses. As CD30 is highly expressed on regulatory FOXP3^+^ T (Treg) cells, we also examined whether CD30 affected the number of Treg cells in aged mice. Remarkably, we found that CD30^+/+^, but not their CD30^−/−^ littermates, exhibited age-dependent T-cell increases in the number of CD4 and CD8 T cells as well as regulatory T cells. This increase in total T-cell numbers was largely due to expansion of memory T cells, with significant effects on numbers of effector memory T cells and a similar trend in the central memory compartment. This may be relevant to the presence of CD30 on expanded T cells in human T-cell lymphoproliferative diseases.

## Materials and Methods

### Mice and Viral Infections

CD30^−/−^ mice (22) generated on the 129 background and extensively backcrossed to C57BL/6 (B6), mice were kindly provided by Tak W. Mak (Ontario Cancer Institute, Toronto). These mice are now available from Jackson Laboratories (Bar Harbor, ME, USA). We analyzed the CD30^−/−^ mice by SNP analysis (performed by The Center for Phenogenomics, Toronto, ON, Canada) and found them to be 96% similar to Charles River B6 mice across 1,200 SNPs. The mice were further backcrossed to B6 mice purchased from Charles River (Wilmington, MA, USA) to generate F2 littermates for experiments. For influenza experiments, male mice (age 5–6 weeks) were immunized with 30 µL of influenza A/PR8 or A/HK-X31 at the indicated doses by intranasal (i.n.) infection while anesthetized with isofluorane. Initial influenza experiments were done in non-littermate mice with all experiments except those at day 100 post-influenza infection confirmed with littermate controls. For PR8 infections, mice were monitored closely with weights monitored daily and were euthanized when moribund. For the chronic infection model, female littermate mice were infected intravenously with 2 × 10^6^ ffu of LCMV clone 13, provided by Michael B.A. Oldstone (Scripps Research Institute, San Diego, CA, USA). All mice were housed in sterile micro-isolator cages under specific pathogen-free conditions. This study was carried out in accordance with the recommendations of the Canadian Council on Animal Care. All animal procedures were conducted as approved by the University of Toronto Animal care committee (animal protocol permit number 200111642).

### *In Vitro* T-Cell Stimulation

Splenocytes from CD30^+/+^ and CD30^−/−^ B6 mice were stimulated *in vitro* with 1 µg/mL of plate-bound anti-CD3 (145-2C11) and 10 µg/ml of soluble anti-CD28 (37.51), and expression of CD30 was assessed by flow cytometry after 24, 48, and 72 h of treatment.

### Flow Cytometry and Intracellular Cytokine Staining

Spleen, mediastinal lymph node (MLN), and lungs were harvested. Lungs were perfused with PBS, and lymphocytes were enriched by isolation over an 80/40% Percoll gradient. Single-cell suspensions were prepared from all organs through mechanical disruption of the tissue with a collagenase digestion step in some experiments and then stained for flow cytometry. MHC class I-peptide monomers were obtained from the National Institute for Allergy and Infectious Diseases tetramer facility (Emory University, Atlanta, GA, USA) and conjugated to streptavidin–allophycocyanin (Prozyme, purchased through Cedarlane, ON, Canada). For intracellular cytokine staining following influenza infection, lung cells and splenocytes were restimulated *ex vivo* with 1 µM of the MHC I-restricted NP_366-74_ peptide for 6 h with GolgiStop (BD Biosciences) at 37°C. For the LCMV experiments, splenocytes were restimulated in the same way with either MHC I-restricted GP_33–41_ or NP_396–404_ peptides or MHC II-restricted GP_60–81_ peptide. Cells were surfaced stained, fixed, permeabilized, and stained intracellularly for specific cytokines as indicated in the figures. Unstimulated samples (no peptide) were used as negative controls. Samples were analyzed using a FACScalibur (BD Biosciences) or LSRFortessa (BD Biosciences) and FlowJo (TreeStar Inc, Ashland, OR, USA) software.

### Antibodies

The antibodies used in this study are as follows: anti-mouse CD30 (clone: mCD30.1) (BD Biosciences, San Jose, CA, USA), anti-mouse CD8α (clone: 53-6.7) (eBioscience, San Diego, CA, USA; BioLegend, San Diego, CA, USA), anti-mouse CD4 (clone: GK1.5) (eBioscience, BioLegend), anti-mouse CD3ε (clone: 145-2C11) (eBioscience, BioLegend), anti-mouse CD44 (clone: IM7) (eBioscience), anti-mouse T-bet (clone: 4B10) (eBioscience), anti-mouse Foxp3 (clone: FJK-16s) (eBioscience), anti-mouse Bcl6 (clone: BCL-DWN) (eBioscience), anti-mouse PD-1 (clone: J43) (eBioscience), anti-mouse CXCR5 (clone:SPRCL5) (eBioscience), anti-mouse CD62L (clone: MEL-14) (eBioscience), anti-mouse IFNγ (clone: XMG1.2) (eBioscience), anti-mouse Tim-3 (clone: RMT3-23) (eBioscience), and anti-mouse CD25 (clone: PC61.5) (eBioscience).

### Focus Forming Assay for LCMV Viral Load

Organs were immediately frozen at −80°C upon harvest. For viral load, organs were thawed and homogenized, and the supernatant collected to perform dilutions (10^0^- to10^5^-fold) for infection of an MC57 cell monolayer under a 2% methylcellulose-MEM overlay. MC57 monolayers were fixed with 4% paraformaldehyde 48 h later, permeabilized with 1% Triton X-100, and stained with Rat anti-LCMV mAb (VL-4). Secondary staining with Goat anti-Rat HRP and *o*-Phenylenediamine (Sigma-Aldrich, Oakville, ON, Canada) was used to induce a colorimetric reaction to label LCMV-infected foci.

### Statistical Analysis

Statistical analysis was performed using GraphPad Prism 6 (San Diego, CA, USA), with the specific test performed as indicated in the figure legends.

## Results

### CD30 Is Expressed on Subsets of T Cells *In Vitro* and *In Vivo*

**Figure 1:**
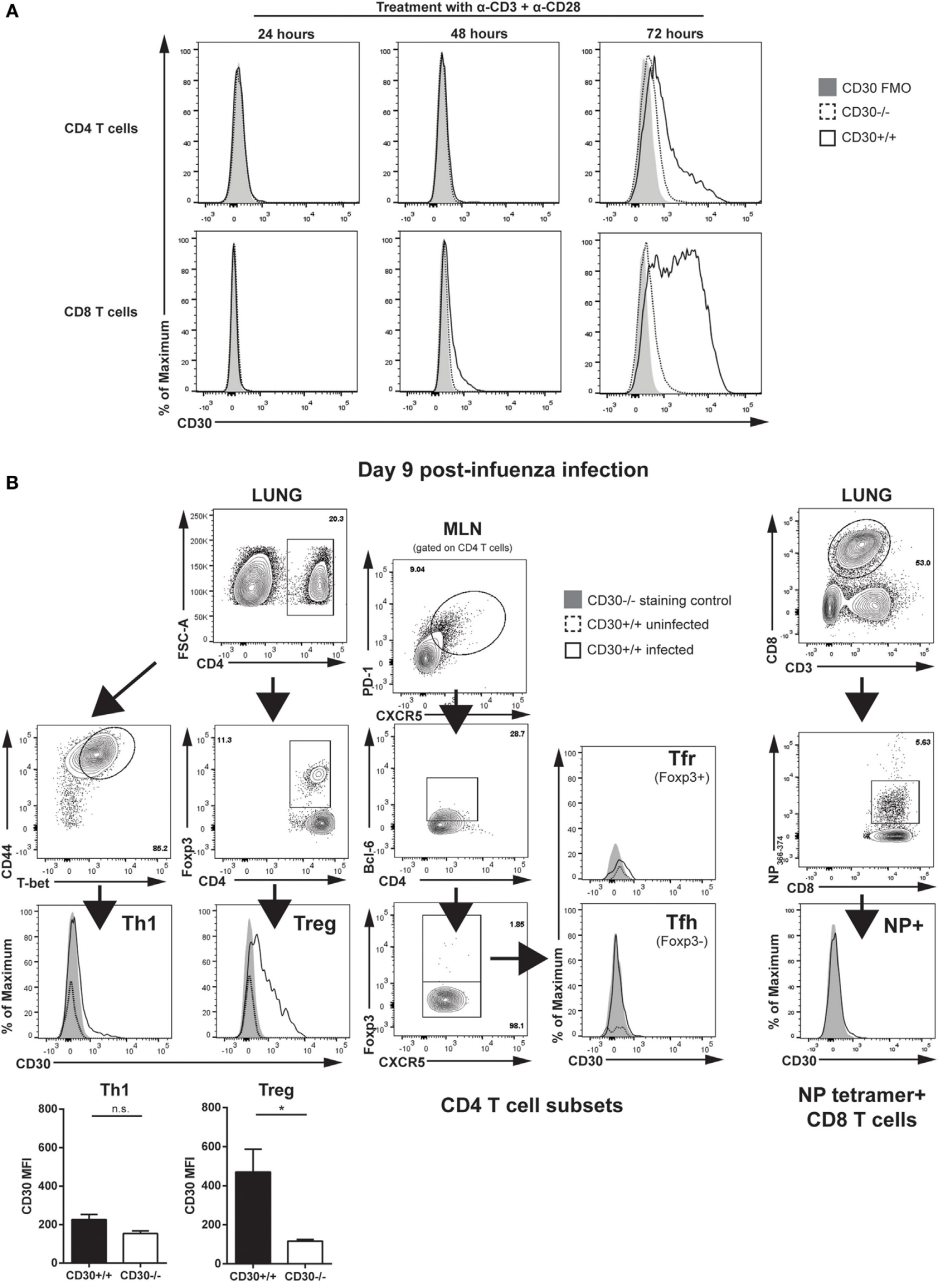
CD30 expression on CD4 and CD8 T cells. **(A)** Splenocytes from CD30^+/+^ and CD30^−/−^ littermate C57BL/6 mice were stimulated *in vitro* with 1 µg/mL anti-CD3 and 10 µg/mL anti-CD28, and expression of CD30 was assessed by flow cytometry after 24, 48, and 72 h of treatment. **(B)** Wild-type CD30^+/+^ mice were infected intranasally with 10^4^ TCID_50_/mouse (sublethal dose) of influenza A/PR8 (PR8), and CD30 expression was assessed on various T-cell subsets in the lung and MLN at day 9 post-infection. Infected CD30^−/−^ littermate mice were used as a staining control. Representative gating strategy and histograms of CD30 expression are shown on D^b^/NP_366-74_ antigen-specific CD8 T cells, T helper 1 (Th1) cells, and T regulatory (Treg) cells from the lung, with T follicular helper cells and T follicular regulatory cells shown from the MLN, with mean fluorescent intensity of CD30 quantified and graphed for Th1 and Treg. Data from **(A)** are representative of two experiments, performed with one mouse per group each experiment, while data in **(B)** are representative of two to three mice per experiment, with two independent experiments performed at day 9 post-infection (median ± range). NS, not significant; *P < 0.05 (Mann–Whitney test).

To examine the expression of CD30 on T cells, we stimulated splenocytes from CD30^+/+^ and CD30^−/−^ littermate mice with anti-CD3 and anti-CD28 antibodies *in vitro*. CD30 was undetectable on resting T cells but induced on CD8 T cells by 48 h of stimulation and on both CD4 and CD8 T cells after 72 h (Figure 1A), consistent with the literature (35). CD30^−/−^ splenocytes were used as a staining control and showed only background levels of staining. Having demonstrated CD30 expression on activated T cells, we next asked whether CD30 could be detected *in vivo*, during a viral infection. Previous work has shown that the inducible TNFR family member 4-1BB is readily detectable on murine T cells in the lung at 6–8 days post-infection with a sublethal dose of influenza A/PR8 (PR8) (27). Therefore, we analyzed CD30 expression at similar time points post intranasal (i.n.) PR8 infection. CD30 was significantly expressed on Treg cells and marginally on Th1 cells but undetectable on antigen-specific CD8 T cells at day 9 pi (Figure 1B). CD30 was not detected at days 3, 5, or 7 in the lung and draining MLN on antigen-specific CD8 T cells, Th1, T follicular helper (Tfh), T follicular regulatory (Tfr), or Treg cells (data not shown, *n* = 2–3 mice per time point). These results confirm that CD30 is expressed during influenza virus infection, albeit on a limited subset of cells, prompting us to examine the effect of CD30 on immunity to influenza virus.

### CD30 Is Dispensable for the Primary Expansion, Memory Conversion, and Secondary Response of Influenza-Specific CD8 T Cell following Acute Respiratory Influenza A Infection

**Figure 2:**
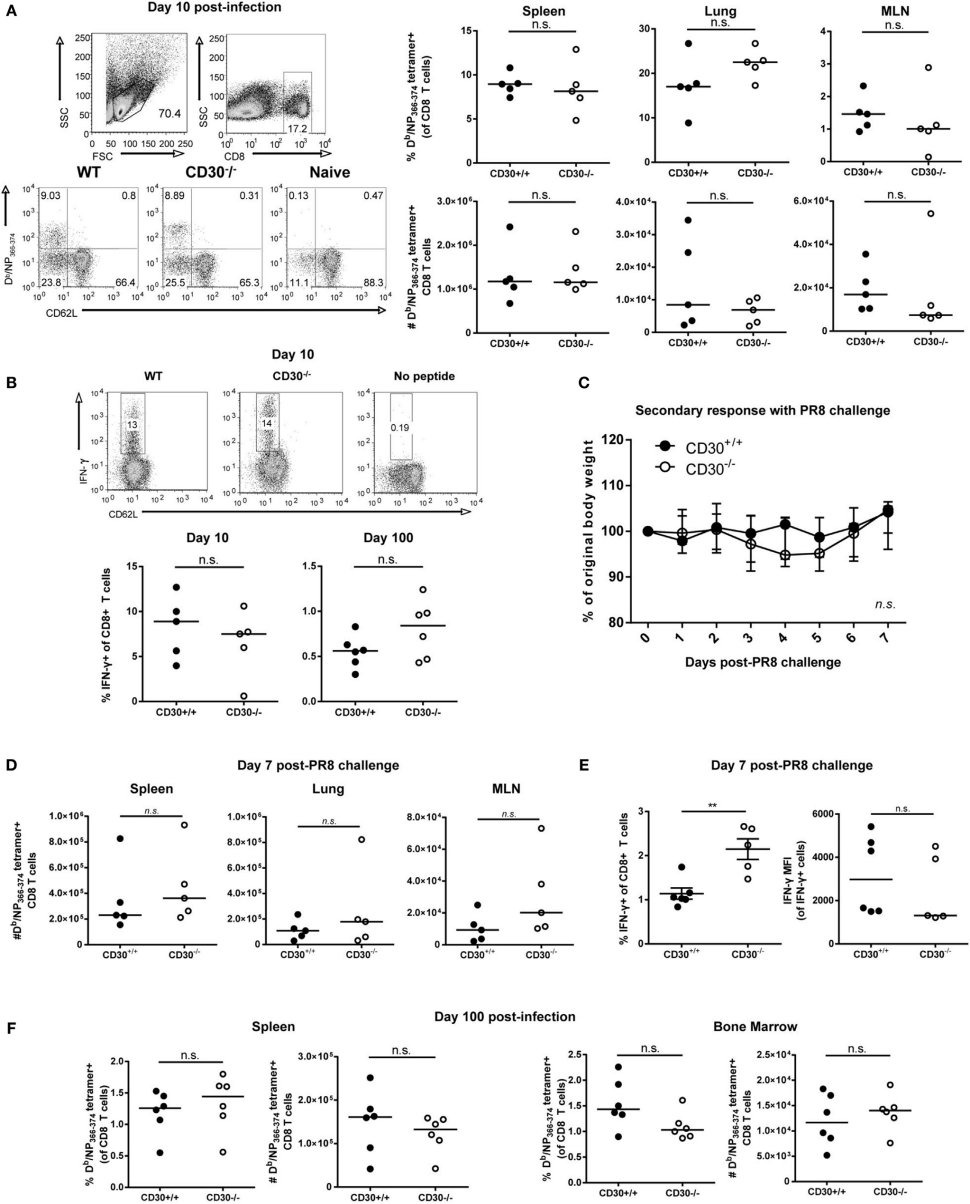
CD30 is dispensable for the primary expansion, memory conversion, and secondary response of influenza-specific CD8 T cells following acute respiratory influenza A infection. CD30^+/+^ and CD30^−/−^ mice were infected intranasally with 5 HAU of influenza A/HK-X31 (X31), and mice were sacrificed at day 10 and day 100 post-infection. The frequency and absolute numbers of D^b^/NP_366-74_ antigen-specific CD8 T cells was determined using MHC tetramers, from various organs at **(A)** day 10 with representative gating. **(B)** Splenocytes from day 10 and day 100 post-infection were restimulated *ex vivo* with NP_366-74_ peptide for 6 h and stained for IFN-γ expression, with representative intracellular staining of IFN-γ-producing CD8 T cells at day 10 post-infection. **(C–E)** CD30^+/+^ and CD30^−/−^ littermate mice were infected with 5 HAU of X31 and then challenged 30 days later with 10^4^TCID_50_ of PR8. **(C)** Weight loss was tracked for 7 days post-challenge. **(D)** The frequency and absolute numbers of D^b^/NP_366-74_ antigen-specific CD8 T cells were determined at day 7, with **(E)** frequency and mean fluorescent intensity of IFN-γ producing CD8 T cells quantified from *ex vivo* NP_366-74_ peptide restimulation of splenocytes. **(F)** The frequency and absolute numbers of D^b^/NP_366-74_ antigen-specific CD8 T cells, from spleen and bone marrow was determined at day 100 after primary X31 infection using fluorescently labelled tetramers. Panel A, B, were done with non-littermate mice but were repeated with littermate mice for PR8 model also with no effect of CD30. Panels **(C–E)** were done with littermate mice. Panel **(F)** was done with non-littermate mice. Data are representative of two (day 100) or three (day 10) independent experiments with *n* = 4–5 per group **(A,B,F)**, and PR8 challenge experiment pooled from two independent experiments with *n* = 2–3 per group per experiment (median) **(C–E)**. NS, not significant; **P < 0.005 (Mann–Whitney test).

In pilot experiments, CD30 was not required for mouse to survive influenza A/PR8 infection and the CD8 T-cell responses to sublethal influenza A/PR8 at day 10 post-infection in CD30^+/+^ and CD30^−/−^ littermates were comparable (data not shown). Therefore, we switched to a milder strain of influenza, Influenza A/HK/X31 (X31, an H3N2 virus) (36), with the idea that a weaker infection might be more costimulation-dependent. CD30^+/+^ and CD30^−/−^ mice were infected i.n. with influenza X31 and, the antigen-specific CD8 T-cell response to the immunodominant epitope NP_366-74_ was assessed using D^b^/NP_366-74_ MHC class I tetramers at day 10 (the peak of the primary CD8 T-cell response). Assessment of the frequency and absolute number of NP_366-74_-specific CD8 T cells in the spleen, MLN, and lung showed comparable primary expansion of influenza-specific CD8 T cells in CD30^+/+^ and CD30^−/−^ mice (Figure 2A). The tetramer^+^ cells were CD62L^lo^ in both groups, indicating an effector phenotype (Figure 2A). Therefore, CD30 is dispensable for primary CD8 T-cell expansion to influenza virus.

*In vitro* studies have shown that CD30 stimulation of T cells can enhance their production of IFN-γ, among other cytokines (6). Therefore, we assessed IFN-γ production by *ex vivo* restimulation with NP_366-74_ peptide at day 10 and day 100 post-X31 infection in CD30^+/+^ and CD30^−/−^ mice and found no significant difference in the proportion of IFN-γ producing CD8 T cells at day 10 or 100 (Figure 2B). Thus, CD30 does not play a discernable role in the function of effector and memory CD8 T cells during influenza infection.

Despite the lack of an obvious role for CD30 in CD8 T-cell responses to influenza A X31, it was possible that CD30 could influence protective memory against a more severe influenza infection, such as induced by influenza A/PR8. To this end, we infected mice with influenza X31 and allowed them to naturally clear the virus and develop a memory response, then challenged the mice at day 30 with a sublethal dosage of influenza A/PR8, which typically causes a 20–25% weight loss in naive mice. PR8 shares the same NP epitope as the initial priming X31 strain but contains different HA and NA proteins (PR8 is H1N1, whereas X31 is H3N2), thereby allowing us to assess protection from CD8 T-cell immunity without the interference of neutralizing antibody responses. CD30^+/+^ and CD30^−/−^ mice exhibited protection to the challenge, losing only about 5% of their body weight before making a full recovery, and exhibited comparable weight loss (Figure 2C). CD30^+/+^ and CD30^−/−^ mice also showed equivalent secondary expansion of NP-specific CD8 T cells in the spleen, lung, and MLN (Figure 2D). IFN-γ production by CD8 T cells upon *ex vivo* peptide restimulation was assessed, and although mean fluorescent intensities (MFIs) were not different, CD30^−/−^ mice had a greater frequency of IFN-γ-producing CD8 T cells than their CD30^+/+^ littermates (Figure 2E), despite disease outcome being comparable. Taken together, these data show that CD30 is dispensable for the establishment and programing of secondary recall responses to influenza virus and does not play a role in the disease outcome of a secondary infection.

A previous study by Nishimura et al. reported that early antigen-specific CD8 T-cell responses to *Listeria monocytogenes* infection in CD30L^−/−^ mice are intact but the memory pool assessed at 84 days post-infection was defective compared to wild-type controls (20). This implies a role for the CD30-CD30L pathway in the generation or maintenance of CD8 T-cell memory. Therefore, we investigated whether CD30 is necessary for the establishment of a long-lived CD8 memory T-cell pool following influenza infection. As the bone marrow is a known reservoir of memory T cells and a preferential organ for CD8 memory T-cell homeostasis, we analyzed this organ as well. Mice were infected with influenza X31 and examined for NP_366–374_-specific CD8 T cells 100 days after infection. There were no significant differences observed between CD30^+/+^ and CD30^−/−^ mice in the spleen and bone marrow at this much later time point (Figure 2F). Therefore, CD30 is dispensable for the generation and maintenance of CD8 memory T cells to influenza virus.

### CD30 Is Dispensable for CD4 T-Cell Responses following Acute Respiratory Influenza A Infection

**Figure 3:**
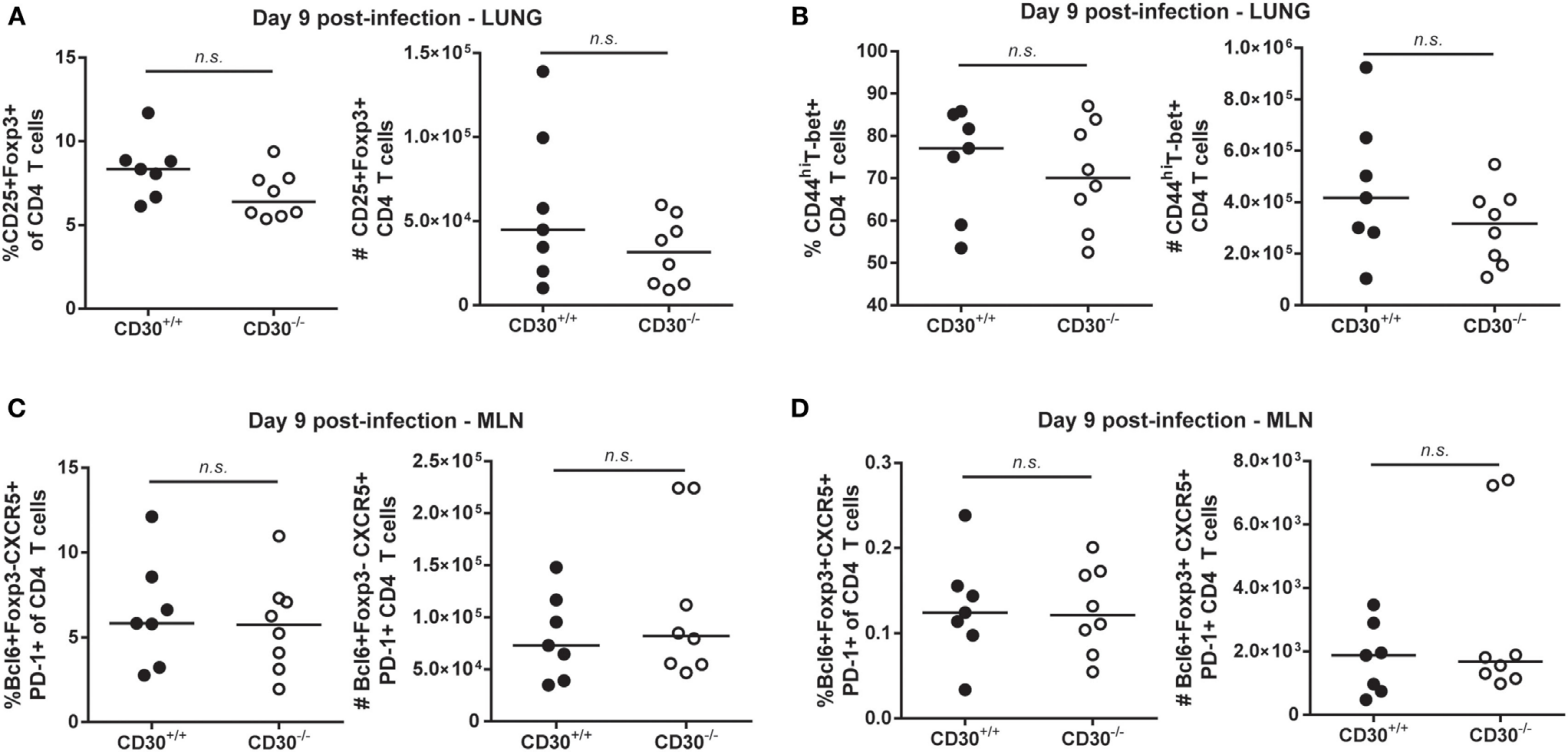
CD30 is dispensable for CD4 T-cell responses following acute respiratory influenza A infection. CD30^+/+^ and CD30^−/−^ littermate mice were infected intranasally with 10^4^ TCID_50_ of Influenza A/PR8, and mice were euthanized at day 9 post-infection. The frequency and number of **(A)** CD25^+^Foxp3^+^ T regulatory cells and **(B)** CD44^hi^T-bet^+^ T helper 1 were evaluated in the lung, and **(C)** Bcl6**^+^**PD-1^+^CXCR5^+^Foxp3^−^ T follicular helper cells and **(D)** Bcl6**^+^**PD-1^+^CXCR5^+^Foxp3^+^ T follicular regulatory cells were evaluated in the mediastinal lymph node. Data are pooled from four experiments with *n* = 2–3 per group per experiment (median). NS, not significant (Mann–Whitney test).

Given the expression of CD30 on lung Treg cells during peak influenza response (Figure 1B), we evaluated the frequency and absolute numbers of Treg cells in CD30^+/+^ and CD30^−/−^ mice at day 9 post-PR8 infection and found no difference in the lung (Figure 3A) despite detecting expression at this time point. Th1 cells, which have only marginal expression of CD30 in the lung (Figure 1B), were also evaluated and comparable populations found as well (Figure 3B). Similarly, no differences in Tfh or Tfr frequencies and absolute numbers were found when the draining lymph node was examined at peak response (Figures 3C,D). Taken together, the data show that CD30 is dispensable for both CD8 and CD4 T-cell expansion during acute influenza infection.

### CD30 Is Dispensable for CD4 and CD8 T-Cell Responses during Chronic LCMV Clone 13 Infection

**Figure 4:**
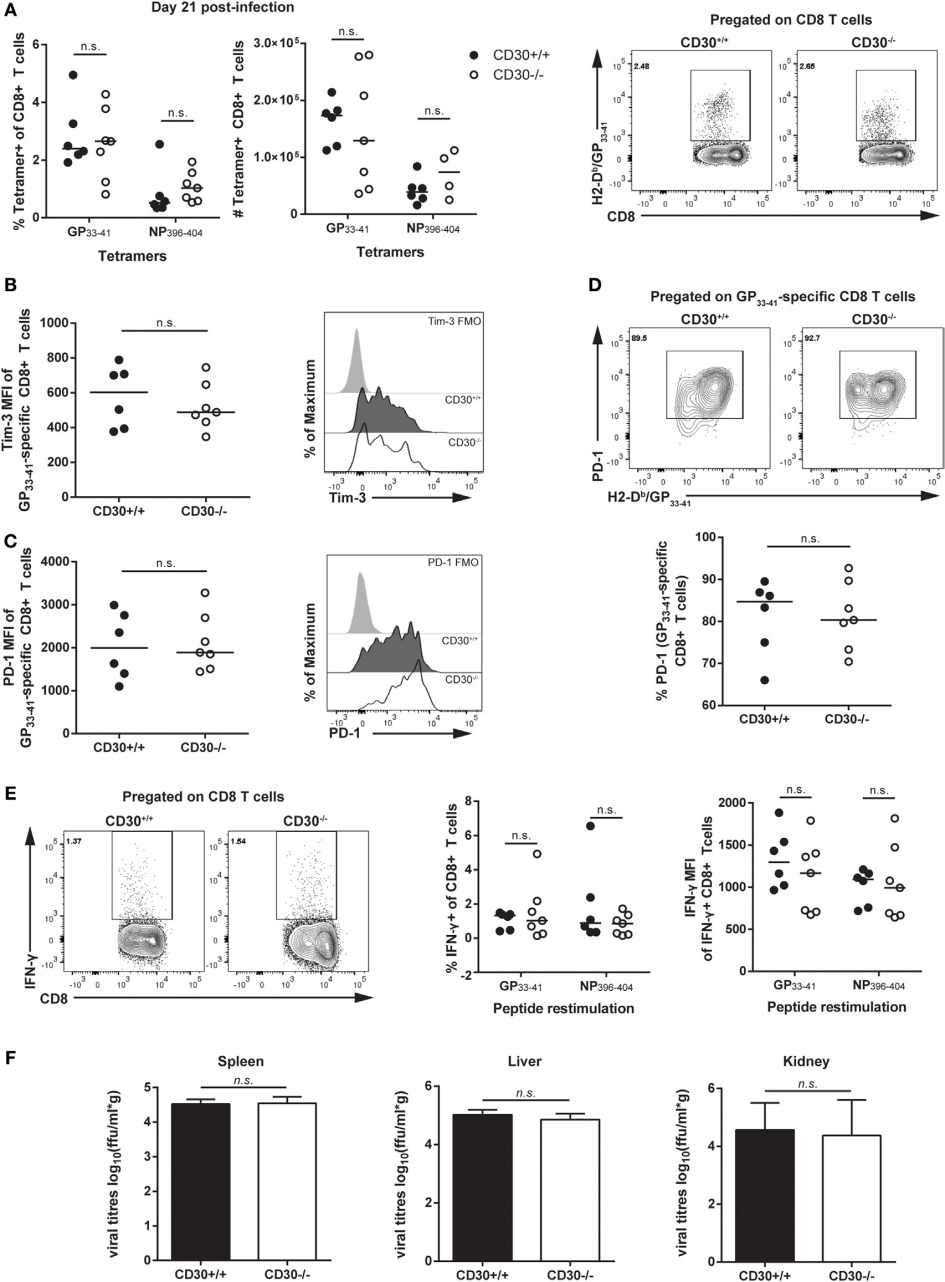
CD30 is dispensable for antigen-specific CD8 T-cell responses and control of LCMV clone 13. CD30^+/+^ and CD30^−/−^ B6 littermate mice were infected with 2 × 10^6^ ffu of lymphocytic choriomeningitis virus clone 13 and sacrificed at day 21 post-infection. **(A)** Splenocytes were isolated and LCMV-specific CD8 T-cell responses were assessed for frequency and absolute number using H-2D^b^/GP_33–41_ and H-2D^b^/NP_396–404_ tetramers with representative staining for the GP_33–41_ tetramer. Mean fluorescence intensity of **(B)** Tim-3 and **(C)** PD-1 within tetramer-specific CD8 T cells were measured along with **(D)** overall frequency of PD-1-expressing tetramer^+^ CD8 T cells (GP_33–41_-specific response is shown, with similar results obtained for NP_396–404_ not shown). **(E)** Splenocytes were restimulated *ex vivo* with GP_33–41_ or NP_396–404_ peptide for 6 h and stained for IFN-γ production, with representative staining shown for GP_33–41_. **(F)** Viral load was evaluated in CD30^+/+^ and CD30^−/−^ mice at day 21 post-infection in the spleen, liver, and kidney. Data are pooled from two independent experiments with *n* = 2–3 per group per experiment (median). NS, not significant (Mann–Whitney test).

It was possible that CD30 is more important in a persistent as compared to an acute rapidly cleared infection. Therefore, we examined the response of CD30^+/+^ and CD30^−/−^ littermates to LCMV clone 13, an infection in which viral clearance takes several months. This chronic infection is characterized by the hierarchical loss of effector functions and peripheral exhaustion of virus-specific T cells (37). We assessed T-cell responses at day 21 post-infection, a time point at which T cells clearly exhibit signs of exhaustion. Frequencies and absolute numbers of antigen-specific CD8 T cells in the spleen, as measured by MHC tetramers containing GP_33–41_ and NP_396–404_ peptides, were comparable between CD30^+/+^ and CD30^−/−^ littermate mice (Figure 4A). Furthermore, the absence of CD30 did not significantly affect the levels of Tim-3 (Figure 4B) and PD-1 (Figure 4C) on the antigen-specific CD8 T cells, two markers whose persistent expression is typically associated with T-cell exhaustion. The overall frequency of PD-1-expressing tetramer^+^ cells was also comparable between CD30^+/+^ and CD30^−/−^ littermates (Figure 4D). *Ex vivo* restimulation of splenic CD8 T cells with GP_33–41_ or NP_396–404_ peptides did not reveal functional differences in their ability to produce IFN-γ, both in terms of total frequencies of IFN-γ-producing CD8 T cells and per cell IFN-γ production as measured by the MFI (Figure 4E). Viral loads assessed in the spleen, lung, and kidney at day 21 also did not differ between CD30^+/+^ and CD30^−/−^ littermate mice (Figure 4F). These results suggest that CD30 is dispensable for the CD8 T-cell response and control of LCMV clone 13 infection.

**Figure 5:**
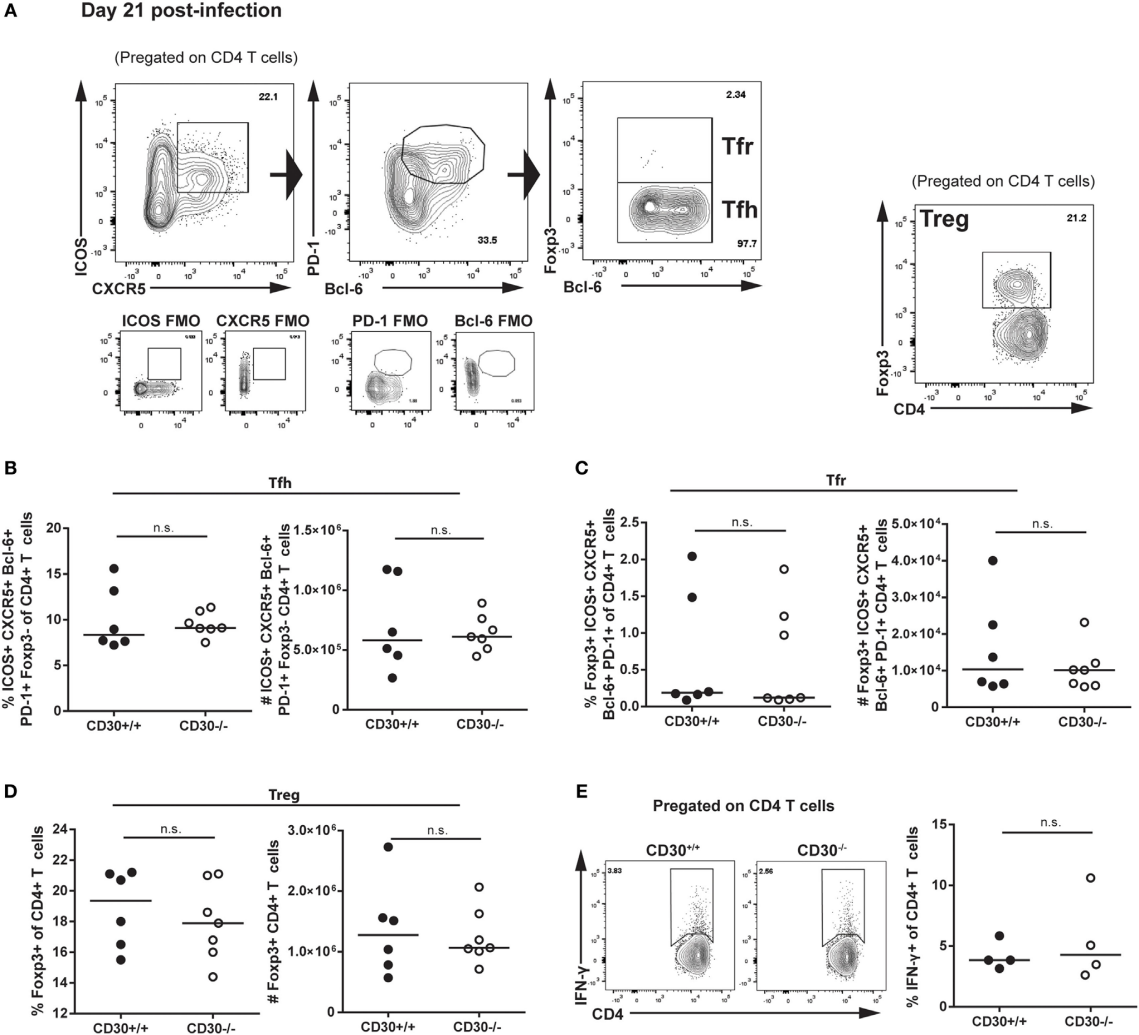
CD30 is dispensable for CD4 T-cell responses during chronic lymphocytic choriomeningitis virus (LCMV) clone 13 infection. CD30^+/+^ and CD30^−/−^ B6 littermate mice were infected with 2 × 10^6^ ffu of LCMV clone 13 and sacrificed at day 21 post-infection. Splenocytes were isolated and examined for CD4 T-cell subsets. **(A)** Gating strategy is shown. Frequency and absolute numbers of **(B)** T follicular helper cells, **(C)** T follicular regulatory cells, and **(D)** T regulatory cells were assessed. **(E)** Splenocytes were restimulated *ex vivo* with the GP_61–80_ peptide for 6 h and stained for IFN-γ production. Representative staining and frequency of IFN-γ-producing CD4 T cells is shown. Data are pooled from two independent experiments with *n* = 3 per group per experiment (median). NS, not significant (Mann–Whitney test).

We next assessed the role of CD30 in the CD4 T-cell response during LCMV clone 13 infection. It was previously reported that mice depleted of CD4 T cells fail to clear the virus, implicating CD4 T cells in control of LCMV clone 13 the infection (38, 39). Analysis of Tfh, Tfr, and Treg populations revealed no difference in frequency or total number of cells (Figures 5A–D). Moreover, *ex vivo* restimulation with the GP_61–88_ peptide did not reveal differences in CD4 T-cell production of IFN-γ, thus suggesting comparable Th1 responses (Figure 5E). Therefore, CD30 is dispensable for CD4 and CD8 T-cell responses in chronic LCMV clone 13 infection.

### CD30 Contributes to the Expansion of the T-Lymphocyte Compartment in Older Mice

**Figure 6:**
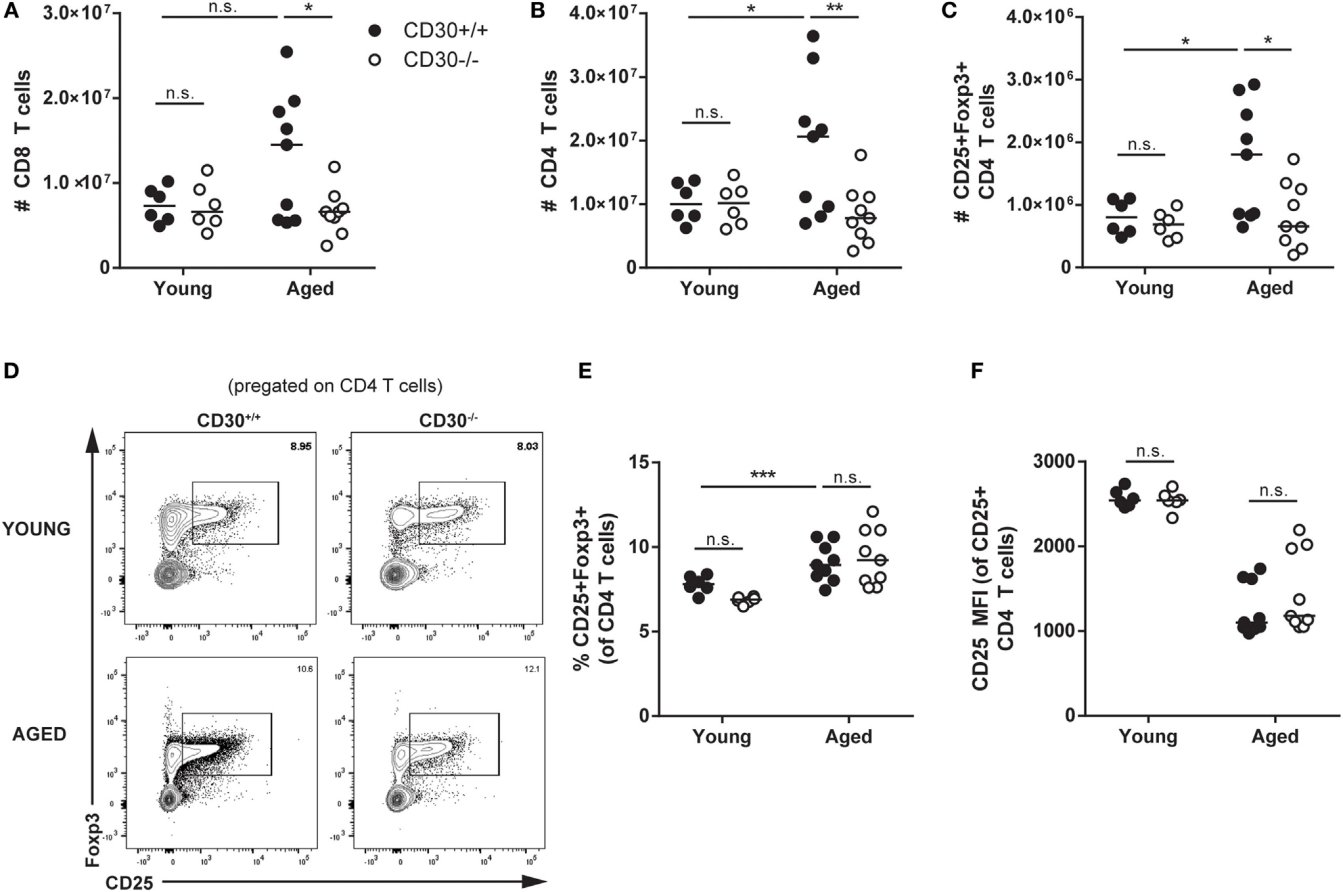
CD30 contributes to expansion of T-cell populations in older mice. Spleens were harvested from young (4–6 weeks) and aged (~8 months) CD30^+/+^ and CD30^−/−^ naive (uninfected) littermate mice and quantified for absolute numbers of **(A)** CD8 T cells, **(B)** CD4 T cells, and **(C)** CD25^+^Foxp3^+^ T regulatory (Treg) cells. **(D,E)** Frequency and representative gating is also shown for Treg cells, with **(F)** CD25 mean fluorescent intensity quantified. Data shown are pooled from three experiments for aged mice and two experiments for young mice, with *n* = 2–4 per group per experiment (median). *P < 0.05, **P < 0.005, ***P < 0.0005; NS, not significant (two-way ANOVA).

As CD30 is expressed at its highest levels on Treg cells during influenza infection and also expressed on T cells in human lymphoproliferative disorders, we asked whether CD30 contributed to the number of steady state T cells, including Treg cells over time. Strikingly, by 8 months of age, wild-type unmanipulated CD30^+/+^ mice had significantly more T cells than their young counterparts (age 4–6 weeks) (Figures 6A–C). While naive young CD30^+/+^ and CD30^−/−^ littermate mice did not show differences in T-cell populations in the spleen, aged CD30^−/−^ mice exhibited significantly fewer CD8 T cells (Figure 6A), CD4 T cells (Figure 6B), and Treg cells (Figure 6C) compared to their wild-type littermate controls. The lower numbers of Treg cells in older CD30^−/−^ mice likely reflects the lower number of CD4 T cells overall, as the frequency of CD25^+^Foxp3^+^ was comparable between CD30^+/+^ and CD30^−/−^ littermates (Figures 6D,E). The MFI for CD25 also did not differ between CD30^+/+^ and CD30^−/−^ littermates (Figure 6F). This age-associated expansion of the T-cell compartment seen in wild-type mice was completely mitigated in CD30^−/−^ littermates, independent of the size and cellularity of the spleens themselves, as the T-cell numbers between young and aged CD30^−/−^ mice were comparable.

**Figure 7:**
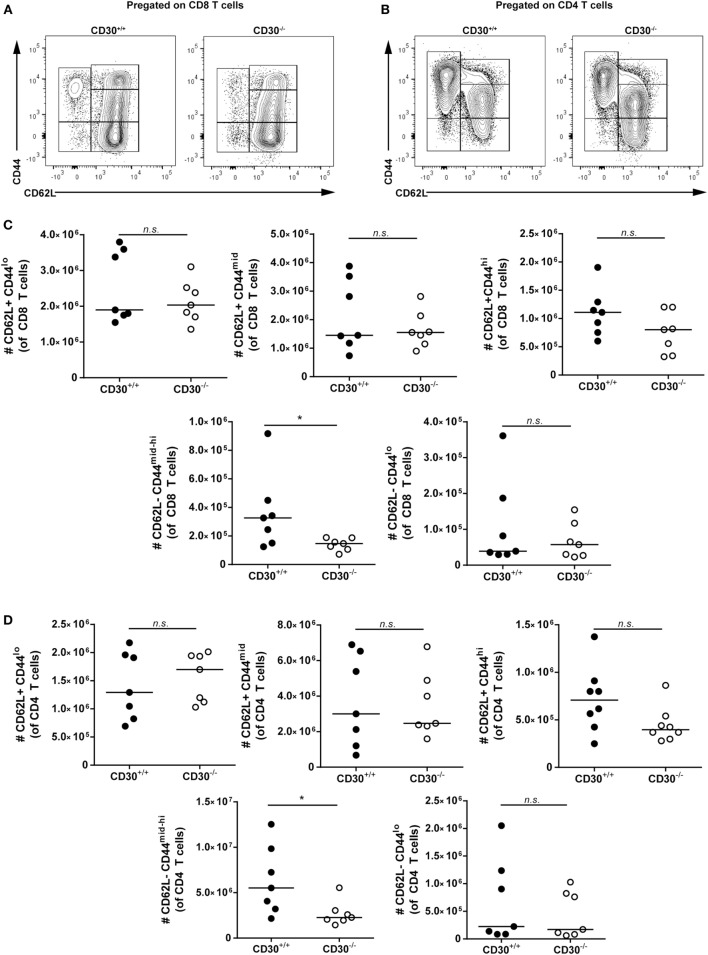
Effector memory but not naive T cells contribute to T-cell expansions in aged CD30^+/+^ mice. Spleens were harvested from aged (~6–8 months) CD30^+/+^ and CD30^−/−^ uninfected spf littermate mice. Representative gating of CD62L^+^CD44^lo^, CD62L^+^CD44^mid^, CD62L^+^CD44^hi^, CD62L^−^CD44^lo^, and CD62L^−^CD44^mid-hi^ subsets of **(A)** CD8 and **(B)** CD4 T cells. Absolute numbers of these subsets are shown in **(C)** CD8 and **(D)** CD4 T cells of aged CD30^+/+^ and CD30^−/−^ littermates. Data shown are pooled from two separate experiments with *n* = 3–4 per group per experiment (median). *P < 0.05; NS, not significant (Mann–Whitney test).

To determine which T cells were expanded in aged CD30^+/+^ compared to CD30^−/−^ mice, we conducted additional analysis of aged littermate mice to differentiate naive and memory T-cell subsets. We assessed CD62L^+^CD44^lo^, CD62L^+^CD44^mid^, CD62L^+^CD44^hi^, CD62L^−^CD44^lo^, CD62L^−^CD44^mid-hi^ subsets of CD4 and CD8 T cells by flow cytometry (Figures 7A–D). CD30^+/+^ and CD30^−/−^ mice had comparable numbers of naive CD62L^+^CD44^lo^ CD8 and CD4 T cells. In contrast, the CD62L^−^CD44^mid-hi^ antigen-experienced effector or effector memory cells were significantly reduced in the knockout mice compared to wild-type littermate counterparts. As KLRG1 was absent from the majority of these T cells (data not shown), the increased T-cell expansions are largely due to memory T cells. This trend was also seen in the CD62L^+^CD44^hi^ central memory T-cell compartment, although it did not reach statistical significance. Thus, CD30 contributes to the expansion of memory T cells in older mice.

## Discussion

Several members of the TNFR family, including OX40, 4-1BB, and GITR, have non-redundant roles in both influenza and LCMV clone 13 infection in mice (27, 30, 33, 34, 40, 41). In contrast, here, we show that the absence of CD30 alone does not appear to impact CD4, CD8, or Treg responses in these two infection models.

We found that CD30 was induced on CD4 and CD8 T cells upon antigen receptor stimulation *in vitro*, consistent with the literature (35). However, *in vivo*, only regulatory T cells expressed high levels of CD30 after influenza virus infection. Th1 cells showed only marginal CD30 expression at day 9 in the lung after infection with Influenza A/PR8, whereas pilot experiments failed to detect CD30 on effector T cells at earlier time points. As TNFR family members are normally transiently upregulated by TCR signaling, it is possible that CD30 is transiently upregulated upon priming of the T cells, but at those time points there are too few responding T cells for us to detect expression immediately *ex vivo*. Nonetheless, based on the evidence that CD30 is expressed on activated T cells, we investigated its role in anti-viral immunity. However, we found no role for CD30 in the T-cell response to influenza or LCMV clone 13. Although we did not exhaustively look at different time points or effector cytokines, the finding that LCMV clone 13 viral load did not change and that lack of CD30 did not affect protective immunity to influenza challenge, as evidenced by mouse survival and ability of CD8 T cells to persist and re-expand, makes it unlikely that CD30 plays a role in the immune response to these viruses.

Of note, a previous study by Nishimura suggested that CD30 was important for the establishment of a long-term central memory T-cell response to *L. monocytogenes*. The differences between our results and those seen by Nishimura et al. may be attributed to the differences in the infections studied. Nishimura et al. showed that CD30L was specifically required for the generation of central memory T cells during infection with *L. monocytogenes*. Even at 100 days post-influenza infection, however, a large proportion of antigen-specific CD8 T cells in WT mice were still CD62L^lo^ (data not shown) and this was particularly evident in the bone marrow. Therefore, the effect of CD30 on CD8 T cells may depend on the type of memory response generated by specific pathogens. It should also be noted that Nishimura et al. (20) did not indicate the use of littermate controls, so it is conceivable that CD30-independent effects of the microbiome on long-term memory could have impacted the results.

Our data showing that CD30 does not play a significant role in the CD8 T-cell responses to influenza and LCMV clone 13 are consistent with that seen with VSV virus infection. In that study, CD30^−/−^ memory CD8 CTL responses were unimpaired and corresponded with similar protection from VSV challenge in both CD30-sufficient and -deficient mice (22). In contrast, another report showed that OX40^−/−^CD30^−/−^ mice had significant defects in antigen-specific CD8 T-cell responses seven days following MCMV infection (21). It should be noted, however, that CD30 single knockout mice were not compared in that model and littermate controls were not mentioned. Moreover, OX40^−/−^ CD30^−/−^ mice did not exhibit any defect in the generation and persistence of CD8 memory T cells. The authors of this manuscript also point out that this may be epitope specific, as has been observed in studies of MCMV in OX40^−/−^ mice (42).

It was previously shown that CD4^+^CD3^-^ accessory cells express high levels of CD30L and OX40L and interact with helper T cells at the T–B border and within B-cell follicles (3, 11). It was therefore possible that the Th2 cells studied at that time were actually the more recently identified Tfh cells. However, we were unable to detect any differences in Tfh or Tfr populations in the two infections studied here. It is possible that during both influenza and LCMV clone 13 infection, CD30 is dispensable and other signals direct follicular helper T-cell differentiation. In fact, Gaspal et al. reported that the defect in germinal centers and antibody production in the absence of CD30 was found only during the memory phase, in sustaining but not forming the germinal centers and in producing memory, but not primary, antibodies (12).

CD30 was initially discovered on neoplastic Reed–Sternberg cells in Hodgkin’s disease, and its overexpression has since been associated with constitutive NF-κB signaling that may promote cell growth and malignancy (43). Its expression has also been characterized in non-Hodgkin’s lymphoma, as well as various cutaneous lymphoproliferative disorders of both T and B cells (44). Here, we found evidence for a role for CD30 in T-cell expansion with aging. CD30^−/−^ mice had lower numbers of total CD4 and CD8 T cells as well as Treg cells at 8 months compared to their wild-type littermates. In contrast, young mice (aged 5–6 weeks) showed no such differences. This difference was due to CD44^mid-hi^CD62L^−^ effector memory T cells, with central memory T cells showing a similar trend. In contrast, naive T-cell numbers were indistinguishable between aged CD30^+/+^ and CD30^−/−^ littermates. CD30, like other members of the TNFR family, induces nuclear factor NF-κB signaling, which may contribute to survival of CD30^+/+^ compared to CD30^−/−^ T memory T cells (45–47).

It is still unclear if the expanded cells are antigen-experienced memory or antigen-inexperienced memory phenotype cells previously characterized in unimmunized mice and in humans (48, 49). Furthermore, whether this effect of CD30 is due to T-cell intrinsic or extrinsic effects remains to be determined. The finding that CD30 plays a role in regulating the size of the memory T-cell pool in aged unimmunized specific pathogen-free mice suggests that in addition to being a marker of T-cell lymphoproliferative diseases, CD30 may contribute to the persistence of T cells over time.

## Ethics Statement

This study was carried out in accordance with the recommendations of the Canadian Council on Animal Care. All animal procedures were conducted as approved by the University of Toronto Animal care committee (animal protocol permit number 200111642).

## Author Contributions

AZ, LS, MW, and TW conceived and designed experiments. AZ, LS, and MW performed experiments. AZ and LS analyzed the data. AZ, LS, MW, and TW wrote and edited the paper.

## Conflict of Interest Statement

The authors declare that the research was conducted in the absence of any commercial or financial relationships that could be construed as a potential conflict of interest.
